# Optimizing trilateration estimates for tracking fine‐scale movement of wildlife using automated radio telemetry networks

**DOI:** 10.1002/ece3.8561

**Published:** 2022-02-10

**Authors:** Kristina L. Paxton, Kayla M. Baker, Zia B. Crytser, Ray Mark P. Guinto, Kevin W. Brinck, Haldre S. Rogers, Eben H. Paxton

**Affiliations:** ^1^ Hawaiʻi Cooperative Studies Unit University of Hawaiʻi Hilo Hawaiʻi National Park Hawaii USA; ^2^ 1177 Department of Ecology, Evolution, and Organismal Biology Iowa State University Ames Iowa USA; ^3^ U.S. Geological Survey Pacific Island Ecosystems Research Center Hawaiʻi National Park Hawaii USA

**Keywords:** automated radio tracking system, localization estimates, movement ecology, radio telemetry

## Abstract

A major advancement in the use of radio telemetry has been the development of automated radio tracking systems (ARTS), which allow animal movements to be tracked continuously. A new ARTS approach is the use of a network of simple radio receivers (nodes) that collect radio signal strength (RSS) values from animal‐borne radio transmitters. However, the use of RSS‐based localization methods in wildlife tracking research is new, and analytical approaches critical for determining high‐quality location data have lagged behind technological developments. We present an analytical approach to optimize RSS‐based localization estimates for a node network designed to track fine‐scale animal movements in a localized area. Specifically, we test the application of analytical filters (signal strength, distance among nodes) to data from real and simulated node networks that differ in the density and configuration of nodes. We evaluate how different filters and network configurations (density and regularity of node spacing) may influence the accuracy of RSS‐based localization estimates. Overall, the use of signal strength and distance‐based filters resulted in a 3‐ to 9‐fold increase in median accuracy of location estimates over unfiltered estimates, with the most stringent filters providing location estimates with a median accuracy ranging from 28 to 73 m depending on the configuration and spacing of the node network. We found that distance filters performed significantly better than RSS filters for networks with evenly spaced nodes, but the advantage diminished when nodes were less uniformly spaced within a network. Our results not only provide analytical approaches to greatly increase the accuracy of RSS‐based localization estimates, as well as the computer code to do so, but also provide guidance on how to best configure node networks to maximize the accuracy and capabilities of such systems for wildlife tracking studies.

## Graphical abstract

The ongoing advancements and innovations of hardware to track animals is generating increasingly powerful systems that can produce unprecedented amounts of data on animal movement. However, analytical approaches for analyzing the resulting data often lag behind technological developments. We present an analytical approach to optimize RSS‐based localization estimates using simple, objective, and efficient methods that greatly increase the accuracy of location estimates. The results of our simulations and evaluation of a field‐based network demonstrate the power of applying analytical filters (signal strength, distance among nodes) prior to RSS‐based trilateration analysis to increase the accuracy of location estimates within a network of nodes.

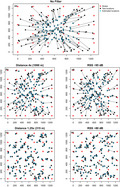

## 1 INTRODUCTION

Advances in electronic tracking technologies have revolutionized our ability to monitor animal movements and behavior over extended periods of time in both terrestrial and aquatic environments. The use of radio, satellite, global positioning system (GPS), and acoustic tracking devices has expanded research on fundamental ecological topics, such as habitat requirements, dispersal, migratory routes, foraging, and home‐range characteristics (McIntyre et al., 2017; Powell et al., 2016; Séchaud et al., 2021; Snijders et al., 2014; Stanley et al., 2021). Each technology has strengths and limitations, and trade‐offs exist for current tracking systems depending on research questions and study species (Bridge et al., 2011). For example, GPS tags are widely used given their accuracy and ability to provide locations anywhere in the world, but their high energy demands require balancing weight of the battery with the lifespan of the tracking device, frequency of location fixes, and the size of the animal that can safely wear the tag. Alternatively, very high‐frequency (VHF) radio telemetry systems utilize lightweight tracking devices that are relatively inexpensive, allowing for large sample sizes on even the smallest of animals (e.g., large insects). However, radio telemetry location data are often restricted to small areas where receivers, which are typically hand‐held devices, can detect signals, limiting the range and time periods over which animals can be tracked. Despite these limitations, radio telemetry remains at the forefront of wildlife tracking studies, particularly for small animals (<30 g), which constitute the majority of flying animals (Bridge et al., 2011).

A major advancement in the use of VHF radio telemetry has been the development of automated radio tracking systems (ARTS), which allow animals to be tracked continuously and simultaneously across potentially large geographic areas. There are different types of ARTS systems, but generally they consist of multiple radio receivers distributed across the landscape detecting radio signals from animal‐borne VHF radios and logging information on the received radio signals for subsequent retrieval and analysis (Kays et al., 2011). Adoption of automated systems has been slow as they are complex systems that are often difficult to construct and optimize, can require considerable up‐front costs to establish, and for which the processing of collected data requires additional analysis with custom computer code. A system that has gained popularity in recent years by overcoming some of these drawbacks is the Motus Wildlife Tracking System (Motus) (Taylor et al., 2017) that provides a platform for linking tracking stations from multiple research groups across large geographic areas (e.g., North and Central America). The collective network uploads received radio signals from animal‐borne radio signals to a central database for researchers to track their study animals across the entire Motus network (Cooper & Marra, 2020; Gómez et al., 2017). Typically, Motus networks are used to track long‐distance movements by detecting when an animal is in the proximity of a receiver, and do not produce high location accuracy. An alternative ARTS system for obtaining accurate animal locations uses multiple directional Yagi antenna mounted on top of elevated towers to generate a bearing to the animal‐borne radio signal from each tower (Larkin et al., 1996). Detections from multiple towers are then used to estimate locations via triangulation (Smetzer et al., 2021; Ward et al., 2013; Zenzal et al., 2018). However, these systems can be complex to set up and optimize, and small errors in bearing estimates can lead to large location errors. A relatively recent approach for monitoring fine‐scale movements of terrestrial wildlife is the use of a network of radio receivers (or nodes) with omni directional antennae that are distributed across a landscape and the radio signal strength (RSS) of a transmitter detected by nodes in the network is used to estimate the animal’s location (Krull et al., 2018; Wallace et al., 2021). A benefit of this approach is the use of relatively simple and inexpensive radio receivers, which has the potential to greatly increase the adoption of this technology, but the use of RSS‐based localization methods in wildlife tracking research is new and still under development.

The approach of using RSS measurements from multiple receivers to estimate a specific location has had widespread use in indoor wireless networks from tracking and monitoring patients in medical facilities to determining the location of packages in a warehouse (Lee & Buehrer, 2019). Signal strength of radio waves decays exponentially with distance, and RSS‐based localizations utilize the relationship between RSS (measured in decibels, dB) and distance from signal source, allowing for accurate localizations in dense networks at small spatial scales (Patwari et al., 2005; Paul & Sato, 2017; Sharma & Prakash, 2018). However, multiple factors can affect the accuracy of RSS‐based localizations, including noise in the environment (e.g., background radio noise, rain, humidity) (Bannister et al., 2008; Luomala & Hakala, 2019) and multipathing effects such as shadowing (i.e., object blocking a signal) or reflection (i.e., signals bouncing off an object), which results in the unpredictable attenuation of RSS values (Liberti & Rappaport, 1992; Whitehouse et al., 2007). These challenges have resulted in a diversity of RSS‐based localization techniques aimed at improving localization accuracy in indoor environments (Kagi & Mathapati, 2021; Yang et al., 2019). However, the application of RSS‐based localization techniques has rarely been applied in structurally complex outdoor environments where vegetation and other structures are prevalent, obstructing or reflecting signals such that RSS values rapidly attenuate, greatly decreasing the range of informative RSS values. Thus, the application of RSS‐based localization to outdoor landscape‐level settings requires development of new analytical approaches that optimize signal‐to‐noise information from a network of nodes to maximize accuracy of location estimates.

In this paper, we build upon the foundations of RSS‐based localization techniques used for indoor wireless networks and develop approaches that optimize RSS‐based localization estimates for node networks designed to track fine‐scale animal movements at landscape scales in structurally complex environments. Specifically, we tested how the application of analytical filters (signal strength, distance among nodes) influenced the error of RSS‐based localization estimates from real and simulated node networks that differed in the density and configuration of nodes. Initial tests of a node network set up to track Såli (Micronesian starlings, *Aplonis opaca*) and brown tree snakes (BTS, *Boiga irregularis*) on the island of Guam indicated that inclusion of information from all nodes in a large network (>70 nodes) provided poor location estimate accuracy (e.g., >200 m localization error) using an RSS‐based approach. We, therefore, developed an analytical approach that objectively identifies and selects nodes that are likely to provide the highest quality information for RSS‐based localizations, and excludes all other nodes from analysis. We predicted that nodes with the highest RSS values reflect those closest to a radio signal source (i.e., animal location), and thus are likely to provide the highest quality information. Therefore, filtering nodes based on RSS values and the distance between nodes would help to remove noise and limit the information used in localization estimates to that which was most accurate. Additionally, we tested whether configuration and spacing of nodes influences localization error. Based on preliminary analysis of data from our Guam network, we suspected that signals in the center of a network had less localization error than signals at the edge, and so we also tested whether the location of a signal within a network affects the error of location estimates. We applied our method to simulated node networks of 100, 175, and 250 m spacing between nodes, a simulated node network with a random spacing of nodes, and test data from our physical network in Guam. The results of this work not only provide analytical approaches to greatly increase the accuracy of RSS‐based localization estimates for tracking the movements of animals at landscape levels, but also the computer code to do so, and guidance on how best to configure node networks to maximize the accuracy and capabilities of such systems for wildlife tracking studies.

## 2 MATERIALS AND METHODS

### 2.1 Guam node network

We established a network of 72 nodes across approximately 226 ha in an urban environment on Andersen Air Force Base on Guam to monitor the movements of Såli and BTS. Each node (CTT Node v. 2, manufactured by Cellular Tracking Technologies, Rio Grande, New Jersey, USA) consists of a small radio receiver with an integrated solar panel and an omni‐directional whip antenna. Nodes continuously scan for uniquely coded IDs emitting from radio transmitters at 433 MHz frequency and log tag ID, signal strength (RSS; range −30 to −120), and a time stamp that is regularly calibrated to GPS satellite time. Nodes periodically (e.g., 30 min) transmit the logged information to base stations (CTT SensorStations, v.2, Cellular Tracking Technologies, Rio Grande, New Jersey, USA) dispersed throughout the node network. Each base station compiles information received from multiple nodes, then transmits the compiled data to internet‐based servers for long‐term storage and remote access. To reduce interference of radio signals with structures in the urban environment, we placed nodes on top of streetlight utility poles, approximately 9 m in height. We attempted to place nodes within 200–250 m of one another but were constrained by the availability and location of telephone poles in the environment. The average distance between a node and the six closest nodes was 216.68 m ± 36.75 SD, range: 160.59 to 358.06 m. While our Guam network used equipment manufactured by CTT, any manufacturer (e.g., Sigma Eight Inc., Lotek Wireless) that offers radio receivers with data logging capability and highly accurate clocks could be used to develop an ARTS node network (e.g., Wallace et al., 2021). Key considerations in selecting nodes for a network design include the accuracy of the clock, consistency of radio receiver sensitivity, along with the ease of communication to a base station or internet‐based server for easy data acquisition, and nodes with low energy demands that can reliably be powered by solar panels.

### 2.2 Establishing the relationship between RSS and distance

To use RSS‐based localization techniques, a relationship between RSS values and distance needs to be established. While a log‐distance propagation model incorporating a path‐loss exponent is the standard approach used to characterize the relationship between distance and RSS values in indoor environments (Dharmadhikari et al., 2018), this approach often results in high localization errors in structurally complex environments where signal bounce and shadowing complicate signal propagation patterns (Lee & Buehrer, 2019). In this situation, regression‐based approaches have shown considerable improvement in characterizing the relationship between RSS values and distance and reducing localization errors (Yang et al., 2011, 2019). Therefore, we utilized an exponential decay function to model the relationship between distance and RSS values. To populate the model, we used information collected from our Guam node network. We measured the signal strength of a radio transmitter at set distances (1, 10, 20, 50, 75, 100, and 125 m) from a random set of individual nodes, ensuring a range of distances (range: 1–2500 m) from all nodes in the network. We attached a coded tag that produces a unique digital signal with a 3‐second pulse interval (CTT PowerTags, Cellular Tracking Technologies, Rio Grande, New Jersey, USA) to a small shampoo bottle filled with liquid (to simulate the mass of a small bird) and then attached the bottle to an approximately 3‐m non‐conductive pole using flagging tape and oriented the transmitter antenna horizontal to the ground to mimic the position of a transmitter on a perched bird. At each test location, we held the pole in a stationary position for a 5‐min time period by placing the bottom of the pole on the ground and orienting the pole so that the transmitter was at a right angle from the node of interest. For each test, we recorded the start and end time and the GPS location in UTMs (WGS84, zone = 55), verifying that the GPS accuracy was <5 m (Paxton et al., 2022).

To process the test data, we downloaded the data from CTT servers and removed the first and last minute of each test to ensure that time stamps between nodes and the GPS unit matched. We then averaged the signal strength values of each node for the 3‐min time period (*n* = 2–59 detections per node) to reduce the influence of outlier RSS values resulting from signal bounce and multipathing. We calculated the Euclidean distance between each node and transmitter test location to determine the true distance between all pairwise combinations of nodes and test locations. We then ran an exponential decay model using a non‐linear least square (nls) approach in R (R Core Team, 2020) to examine the relationship between recorded RSS values and the true distance of the node from each test location:

(1) $$\text{RSS}∼a*\exp\left(‐S*\text{distance}\right)+K$$

where *K* = horizontal asymptote of RSS values, *a* = intercept, *S* = decay factor. The model was first run including the self‐starter function ‘SSasymp’ to help determine starting values for parameters in the final model. To avoid overfitting the model and to allow flexibility in the application of the model output for localization estimates in a dynamic network where nodes may change (e.g., node failure, expanding network area), we did not include additional explanatory variables in the model, such as node identification to account for node variability. However, researchers can calibrate nodes prior to deployment to account for between‐node variability in detection sensitivity of RSS values, which may reduce some error (Bircher et al., 2020).

### 2.3 RSS‐based localization estimates and test datasets

To estimate locations, we used an RSS‐based trilateration (multilateration >3 nodes) approach described in Lee and Buehrer (2019). Trilateration uses the signal strength received by a node and the exponential relationship between RSS values and distance described in Equation 1 to estimate distance of each node to the signal source. In a geometrical sense, the estimated distance represents the radius of a circle centered at the node with the circumference of the circle defining the range of possible locations of the source signal. Theoretically, when three or more nodes detect a signal at the same time, the common intersection of the circles represents the location of the signal, but factors such as noise, signal bounce, and obstructions prevent perfect convergence. We, therefore, implemented the trilateration approach using a non‐linear least squares model (‘nls’ function in program R) (R Core Team, 2020) that uses a Gauss–Newton algorithm to optimize the location estimate of a received signal. The model minimizes differences between pairwise Euclidean distances of all nodes and the estimated distance of the signal from each node. We specified starting location values for the model based on the location of the node with the strongest signal, and estimated distance of the signal from each node based on the average RSS to distance relationship (Equation 1) from simulated or true RSS values received by each node.

To assess the influence of node spacing on the error of RSS‐based localization estimates, we simulated three node configurations within a 12.5 km^2^ grid. Nodes were evenly distributed within the grid with distances between nodes at 100 m (*n* = 100 nodes), 175 m (*n* = 64 nodes), and 250 m (*n* = 36 nodes). Additionally, to understand whether a non‐uniform, random distribution of nodes within a network influences localization error, we created thirty‐six 250 m^2^ grids within the 12.5 km^2^ grid and randomly placed a node anywhere within each grid.

We then tested how grid spacing (distance among nodes), regularity (consistency of spacing among nodes), and location within a node network (distance from edge) affects the error of RSS‐based localization estimates. We first created a set of 100 random points within the 12.5 km^2^ simulation grid to use as test locations, and then calculated the true distance between the 100 test locations and each node in the network for each simulated node configuration. We then generated an RSS value for every paired node and test location, per simulation, based on our Guam network’s relationship between RSS and distance. Specifically, we used the true distance between a test location and a node to randomly select an RSS value associated with that distance in our Guam test dataset. This process allowed us to incorporate variability in signal strength values for a given distance based on noise in our Guam network. We repeated this step 1000 times for each test location (*n* = 100) creating 100,000 simulations per node configuration. To compare simulated results with a real‐world dataset, we randomly selected 54 locations within our Guam network and measured the signal strength of a stationary radio transmitter from each node in our network using the same procedures as described above (Paxton et al., 2022).

### 2.4 Location estimation filters

We used two filtering approaches prior to RSS‐based localization estimates. The first was a simple RSS filter, where only nodes that received an RSS signal above a certain threshold were included in the trilateration analysis. We tested RSS cutoff values of −80, −85, −90, and −95 (dB) to provide a reasonable range of filters to assess the balance between location accuracy and data loss. The relationship between RSS values and distance is stronger at higher RSS values, and thus, error is expected to decrease with more stringent RSS filters. However, trilateration requires at least three nodes to estimate a location, so as the RSS filter becomes more stringent, the number of locations that meet the criteria (i.e., ≥3 nodes with RSS values above the filter level) will likely decrease for some network configurations. In most cases, the nodes receiving the strongest RSS values are expected to be the nodes closest to the transmitter, but given a real‐world landscape with structures and vegetation that can cause bounce and attenuation of the signal strength, an RSS filtering approach may not necessarily ensure that the closest nodes are chosen. Therefore, we assessed a second approach where we identified the node receiving the strongest RSS value, assumed to be the node closest to the radio transmitter, and then selected all nodes within the network that were within some distance of the node receiving the strongest RSS signal. For this analysis, we tested distance filters that were a multiple of the average grid spacing (i.e., 1.25×, 2×, 3×, and 4× distance of the average network spacing) to account for networks with different node spacing.

We evaluated the ability of filters to decrease location estimation error by applying the filters described above prior to conducting each trilateration analysis. Localization error of trilateration location estimates was assessed by calculating the difference in distance (m) between the true and estimated location with lower values indicating higher accuracy of localization estimates. We also evaluated the role that location within a network played in location estimation error by examining the relationship between error of estimated test locations and the distance of each test location to the nearest edge of the network.

All simulations and analyses were conducted in the R Statistical Software Environment version 4.0.3 (R Core Team, 2020).

## 3 RESULTS

**FIGURE 1:**
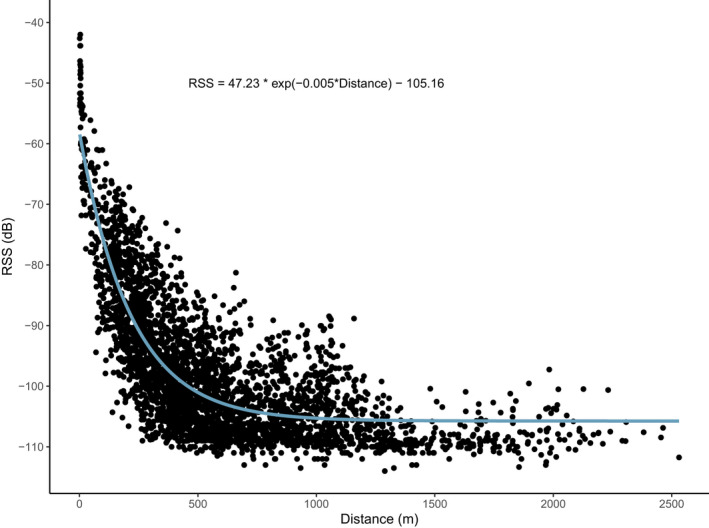
Negative exponential decay model showing the relationship between receiver signal strength (RSS) values and the distance of a test transmitter from each node based on 135 random locations of a test transmitter within a network of 72 nodes on Guam. The equation RSS = 47.23*exp(−0.005*Distance) − 105.16 describes the exponential decay of RSS values with each unit of change in distance

A test dataset of 135 known radio transmitter locations distributed throughout the Guam network resulted in 3390 data points with RSS values and associated known distances to nodes in the network. Modeling a negative exponential decay model (Equation 1) to the dataset showed a rapid decline in RSS values as the distance between nodes and the test transmitter increased (Figure 1), with the relationship expressed as:

$$\text{RSS}=47.23*\exp\left(‐0.005*\text{distance}\right)‐105.16$$

**FIGURE 2:**
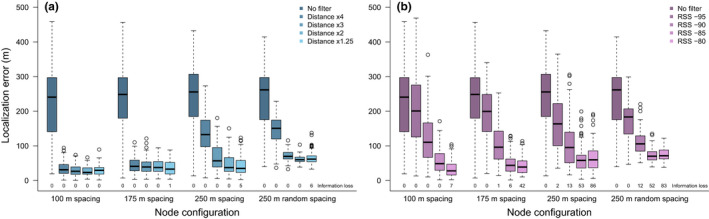
Boxplots showing localization error (difference in meters between the true and estimated location) of trilateration location estimates for simulated node configurations that vary in the density of nodes (100, 175, 250 m) and consistency of spacing among nodes (uniform, random). Prior to trilateration, we applied either no filter, distance‐based filters (a) which included only nodes within a specified distance of the node that received the strongest radio signal, or receiver signal strength (RSS)‐based filters (b) which included only nodes that received radio signals greater than the defined RSS value. Additionally, the percentage of locations that could not be estimated (information loss) is indicated below each boxplot. Boxplot whiskers depict the 10th and 90th percentiles and boxes show the 25th and 75th percentiles with the median value indicated, outliers shown as circles

**FIGURE 3:**
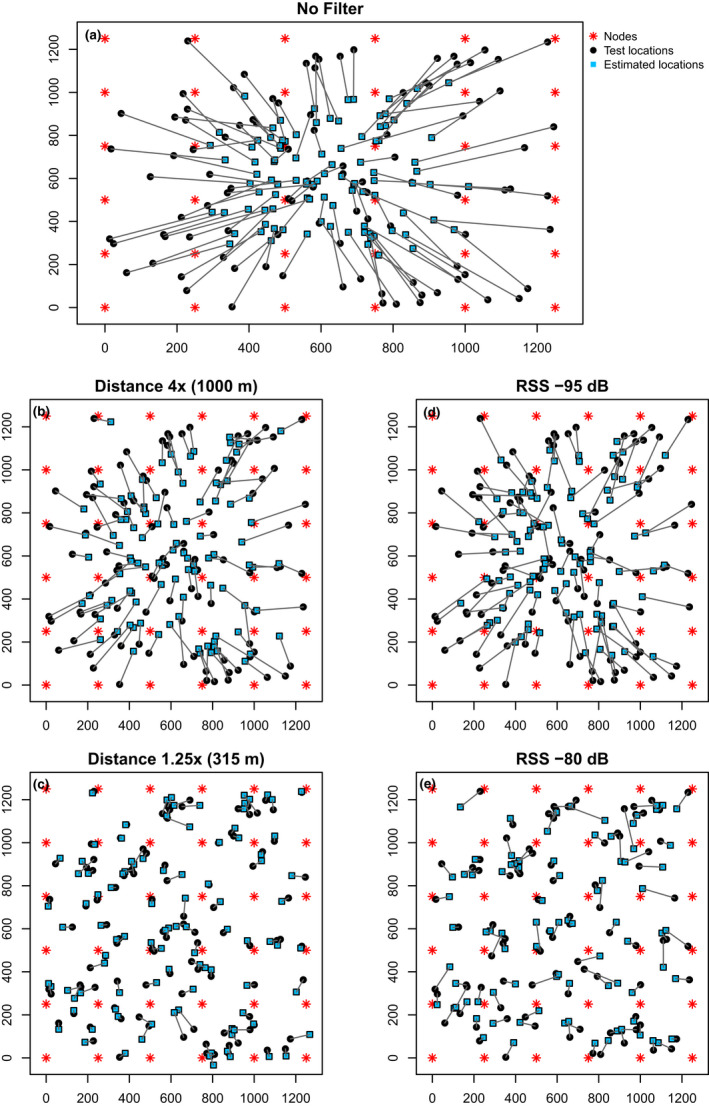
Representation of the 250 m simulated node configuration with 36 nodes (red asterisks) uniformly spaced within a 12.5 km^2^ grid, along with the 100 random test locations (black circles) each connected by a line to their estimated location (blue squares) based on trilateration. Prior to trilateration, we applied either no filter (a), distance‐based filters using only nodes within 1000 m (b) or 315 m (c) of the node that received the strongest radio signal, or receiver signal strength (RSS)‐based filters using only nodes that received radio signals greater than −95 dB (d) or −80 dB (e)

The error of location estimates using trilateration of all data (i.e., no filter) was high for all simulated node configurations and the Guam network test data, with the median difference between true and estimated locations ranging from about 172 to 249 m (Figure 2, Table 1). The inclusion of all nodes in a network for trilateration estimates tended to pull location estimates to the center of a network (Figure 3), indicating that estimated locations were primarily an average of all the node locations included in the analysis.

**TABLE 1:** Summary of localization error (difference in meters between the true and estimated location) of trilateration location estimates for a field‐based node network on the Island of Guam and simulated node configurations that vary in the density of nodes (100, 175, 250 m) and consistency of spacing among nodes (uniform, random)

Filter	% Location loss	Mean no. nodes	Mean error	Lower 95% CI error	Upper 95% CI error	Median error	Min error	Max error
Nodes 100 m uniform spacing								
No Filter	0	112.82	222.58	203.12	242.05	240.62	19.34	458.70
Distance 4× (400 m)	0	36.28	34.30	30.71	37.90	30.97	1.12	94.21
Distance 1.25× (125 m)	0	4.75	29.46	26.37	32.55	29.34	1.53	89.36
RSS −95 dB	0	39.79	199.75	180.92	218.58	200.76	13.10	469.07
RSS −80 dB	1	8.33	33.54	28.95	38.12	27.86	2.23	104.03
Nodes 175 m uniform spacing								
No Filter	0	41.65	242.73	224.88	260.57	248.43	7.09	456.74
Distance 4× (700 m)	0	25.28	43.56	39.23	47.88	40.71	3.30	110.15
Distance 1.25× (220 m)	0	4.59	36.37	32.22	40.51	32.77	3.53	88.16
RSS −95 dB	0	14.22	194.28	179.39	209.16	199.39	20.10	340.47
RSS −80 dB	42	3.61	42.72	38.10	47.34	38.78	10.06	113.68
Nodes 250 m uniform spacing								
No Filter	0	23.19	238.99	221.04	256.94	255.53	13.62	432.60
Distance 4× (1000 m)	0	19.49	137.34	126.70	147.98	132.55	7.78	273.28
Distance 1.25× (315 m)	0	4.39	41.87	36.37	47.36	34.80	2.74	123.49
RSS −95 dB	0	7.53	167.08	152.47	181.69	163.36	35.36	365.02
RSS −80 dB	86	3.07	67.69	59.10	76.27	59.53	5.75	191.36
Nodes 250 m random spacing								
No Filter	0	22.77	239.07	222.36	255.78	249.35	23.03	462.61
Distance 4× (1000 m)	0	19.34	144.46	136.27	152.64	140.93	17.30	320.36
Distance 1.25× (315 m)	5	4.42	64.73	61.39	68.25	55.10	4.72	276.78
RSS −95 dB	0	7.50	170.76	159.61	181.91	167.80	19.13	375.78
RSS −80 dB	83	3.10	74.61	71.54	78.34	66.93	9.75	221.02
Guam nodes ~215 m random spacing								
No Filter	0	22.83	180.77	155.08	206.45	171.57	24.38	417.06
Distance 4× (1000 m)	0	20.98	141.94	125.02	158.85	140.05	16.90	285.12
Distance 1.25× (315 m)	0	7.37	82.69	68.20	97.19	73.19	4.94	266.35
RSS −95 dB	0	11.44	115.19	91.95	138.42	85.29	14.51	345.86
RSS −80 dB	40	3.72	62.23	47.16	77.29	49.85	11.27	167.60

Notes

Prior to trilateration, we applied either no filter, distance‐based filters which included only nodes within a specified distance of the node that received the strongest radio signal, or receiver signal strength (RSS)‐based filters which included only nodes that received radio signals greater than the defined RSS value. Only results for no filter and the least and most stringent RSS and distance filters for each node configuration are shown. Additionally, the mean number of nodes included in trilateration estimates is indicated, along with the percentage of locations that could not be estimated with trilateration (location loss).

Filtering data prior to trilateration using either an RSS value or distance filter greatly reduced the error of location estimations (Figures 2 and 3). Filters resulted in a subset of nodes being used in the trilateration analysis (Table 1), localizing estimates to a restricted portion of the network, and decreasing localization error for all simulated node configurations and the Guam network test dataset as filters became more stringent. The median difference between true and estimated locations for the most stringent filters (i.e., Distance 1.25×, RSS −80) ranged from about 29 to 73 m, a 3.4‐ to 8.6‐fold decrease in localization error from unfiltered trilateration estimates (Figure 2, Table 1).

The error of location estimates increased as the distance between nodes in a network increased, even with stringent filters, such that the 100 m node configuration had the lowest localization error, followed by the 175 and 250 m node configurations. Non‐overlapping 95% confidence intervals (CI) indicated that differences in localization error of most filters were significant between node configurations (Figure 2, Table 1). In addition, less uniform spacing of nodes in a network also increased localization error, with a non‐uniformly spaced 250 m simulated network having 1.6 times more localization error than a uniformly spaced 250 m simulated network when using the most stringent distance filter (Figure 2, Table 1).

Node configuration influenced whether the application of RSS or distance filters resulted in less localization error. For example, the 100 and 175 m node configurations had no difference in the average error of the most stringent RSS and distance filters (based on overlapping CI; Table 1). However, for the 250 m node configuration, the most stringent distance filter, on average, provided less error of location estimates compared to the most stringent RSS filter (based on non‐overlapping CI; Table 1). Moreover, 86% of simulated locations could not be estimated when the most stringent RSS filter was applied, compared to only a 5% loss of simulated location estimates for the most stringent distance filter (Table 1). Overall, the distance‐based filters had little to no loss of location estimates. However, the relative decrease in localization error of a distance‐based versus RSS‐based filter diminished when the spacing of nodes was irregular (i.e., 250 m random spacing node configuration). In a test of our Guam network, which had very irregular spacing among nodes, the most stringent RSS filter, on average, had 20 m lower localization error than the most stringent distance filter, although there was considerable overlap in their CI (Table 1).

**FIGURE 4:**
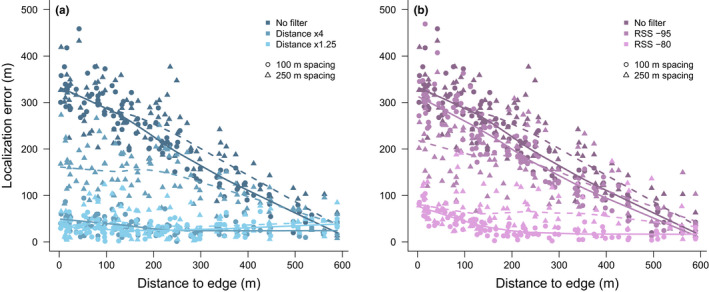
Relationship between the distance of each test location from the nearest edge of the network and localization error (difference in meters between the true and estimated location) of trilateration location estimates for simulated networks with uniformly spaced nodes every 100 m (circles) or 250 m (triangles). Prior to trilateration, we applied either no filter, distance‐based filters (a) which included only nodes within a specified distance of the node that received the strongest radio signal, or receiver signal strength (RSS)‐based filters (b) which included only nodes that received radio signals greater than the specified RSS value. Loess lines were fit to the relationship between accuracy and distance to edge for each filter and node configuration (solid lines for 100 m, dashed lines for 250 m)

The location of a radio signal within a network strongly influenced localization error when no filters were applied. Generally, localization error increased as the distance of a radio signal to the edge of a network decreased (Figure 4). The average localization error of a signal 600 m from the edge of a simulated network was less than 50 m, whereas the average localization error for a signal on the edge of a network was ~300 m. However, the application of both RSS and distance filters prior to trilateration analysis greatly reduced the error of location estimates associated with signals near the edge, and overall flattened the relationship between error and distance from edge (Figure 4), although the application did not entirely remove all edge effects.

## 4 DISCUSSION

The ongoing advancements and innovations of hardware to track animals is generating increasingly powerful systems that can produce unprecedented amounts of data on animal movement. Approaches for analyzing the resulting data are critical for extracting the highest quality and quantity data possible; however, analytical approaches often lag behind technological developments. We present an analytical approach to optimize RSS‐based localization estimates using simple, objective, and efficient methods that greatly increase the accuracy of location estimates. The results of our simulations and evaluation of a field‐based network demonstrate the power of applying node‐excluding filters prior to RSS‐based trilateration analysis to reduce the error of location estimates within a network of nodes. Our approach helps to minimize inherent limitations of localization algorithms in estimating locations in noisy, outdoor environments to track fine‐scale animal movements. Overall, the use of signal strength and distance‐based filters resulted in an approximately 3‐ to 9‐fold decrease in median error of location estimates over unfiltered estimates, with the most stringent filters providing location estimates with a median localization error ranging from about 29 to 73 m depending on the configuration and spacing of the node network. In addition, our simulations of node networks that varied in the density and regularity of node spacing provide insights into how node configuration influences accuracy of location estimates, important information that researchers would need to consider when designing effective networks in different environments and for different research questions.

Our work clearly shows that inclusion of information from all nodes in a network for RSS‐based localization estimates does not improve the accuracy of location estimates, at least in noisy outdoor environments. In fact, the most stringent filters, which produced the lowest errors in location estimations, included RSS information from the least number of nodes. The inclusion of signal strength information from all nodes in a large network creates challenges for RSS‐based localization estimates because radio signals quickly attenuate at increasing distances from a transmission source, resulting in the relationship between RSS and distance rapidly degrading (Whitehouse et al., 2007). Thus, many of the nodes farther from a radio signal source will have low RSS values that are poorly correlated with geographic distance, increasing the probability of introducing outlier distance estimates into localization algorithms, and creating high uncertainty in location estimates (Li et al., 2014). This results in estimated locations being more reflective of the node locations included in the analysis rather than the actual animal location, and shifts estimated locations toward the center of the network. When no filters are applied, the inclination of the trilateration algorithm to estimate locations at the center of the network is clearly illustrated in Figure 3 (no filter) and is likely the cause for the strong edge effect observed with non‐filtered trilateration estimates. Thus, location estimates from only a subset of nodes help constrain the area within the network for localization and use only the highest quality information for the trilateration estimations.

A key goal of this research was to develop an objective approach to remove nodes with low‐quality information that can be automatically applied to all localization estimates, particularly given the need to process large quantities of data generated from these types of node networks (e.g., signal detection every 1–15 s, depending on tag programing specifications). For example, a telemetry tag that emits a pulse every second would have up to 86,400 potential localization estimations per 24 h. The use of RSS values as a filter is a simple and intuitive approach given the known relationship between accuracy of distance estimates and RSS values. However, we found that a distance‐based approach was more powerful in our simulations than an RSS‐based approach. A distance‐based approach likely had lower localization error because a distance filter maintains a configuration of all nodes around the location to be estimated, better balancing the optimization approach utilized by RSS‐based localization algorithms. Moreover, the use of a distance‐based approach resulted in little to no reduction in the number of locations that could be estimated because when nodes are evenly spaced this approach typically maintains the minimum of three nodes needed for trilateration. In a grid with nodes spaced 250 m apart, there were, on average, four nodes included in the trilateration analysis when the most stringent distance filter (i.e., 1.25×) was applied, while a 2× distance filter had an average of 9 nodes included in localization estimates. Even if some of the nodes maintained by the distance filter have low RSS values, their influence is localized to the neighborhood of interest, and thus does not skew location estimates to other regions of the network. In contrast, the use of a minimum RSS filter does not always maintain a configuration of nodes that surrounds the neighborhood of an unknown location, nor does an RSS filter necessarily retain a minimum number of nodes. In the simulations for a grid with nodes spaced 250 m apart, 85% of locations could not be estimated (i.e., <3 nodes) with the most stringent RSS filter (−80 dB), and of those locations that could be estimated, the average number of nodes was just above 3 nodes.

While a distance filter performed significantly better than an RSS filter in networks with evenly spaced nodes, the advantage diminished when nodes were less uniformly spaced within a network. The simulated 250 m uniform grid had an approximately 1.6‐fold (26 m difference in accuracy) lower localization error for distance‐based versus RSS‐based filters, but for the 250 m random grid the advantage was only approximately 1.2‐fold lower (10 m difference in localization error). Applying filters to test data generated in our Guam node network, which has uneven spacing of nodes, resulted in the RSS filter actually performing better, on average, than the distance filter. However, overlapping CI between the most stringent distance and RSS filters indicated that the difference in localization error was small (CI RSS −80: 47.16–77.29 m; CI Distance 1.25×: 68.20–97.19 m). The reduced advantage of distance‐based filters for node networks with uneven spacing may be due to the optimization of the trilateration algorithm, where uneven spacing of nodes adds additional noise to the system, thus reducing the accuracy of location estimates (Bhat & Santhosh, 2020).

The relationship between RSS and distance will likely differ among environments and influence the accuracy of location estimates. While indoor wireless sensor networks using RSS‐based localization estimates can provide near GPS‐level accuracy (Lee & Buehrer, 2019), outdoor environments are complex, and many factors can degrade the accuracy of RSS‐based localization estimates, including the distances from which signals are detected, the height that nodes can be deployed, overall levels of background noise, and the structure of the environment in terms of objects or vegetation that will potentially cause obstruction or signal bounce (Whitehouse et al., 2007). Moreover, the behavior and spatial orientation of the focal species of interest will influence the error of localization estimates. For example, the signal from a bird that spends most of its time at the top of tall trees will likely have less attenuation of signal strength with distance than a signal from a snake that is on the ground in dense understory (Rutz et al., 2015). All of these factors will influence the optimal spatial configuration of nodes needed for a given environment and study species. Additionally, our simulations highlight the importance of considering edge effects when designing a network, given that error of localization estimates increased near network edges. Identifying the key areas where researchers want to collect data and ensuring those areas are in the core area of a network, and not at edges, will increase accuracy of location estimates. Therefore, it is important that the configuration of a network is carefully planned prior to deployment of nodes with consideration given to the level of resolution of tracking data needed to address the objectives of a research project. We recommend researchers first conduct testing with a reduced configuration of nodes in their study area to quantify the relationship between RSS and distance within their environment. Researchers can then conduct simulations of alternative network configurations utilizing their RSS to distance relationship to identify an optimal design for their study system, and evaluate which filters provide the lowest localization error for their network. The methods for simulating and testing node networks outlined in this paper, along with the R code to simulate and process data, provide tools that researchers can use to optimize networks for trilateration localization methods.

RSS‐based localization estimates for node networks tracking animal movements are attractive for their simplicity, with the only information needed being the location of nodes, signal strength of a received radio signal, and the time that a signal is received. Thus, using an RSS‐based localization method requires minimal hardware (Zekavat et al., 2019), allowing for relative ease of deployment of large multi‐node networks with minimal upfront costs to simultaneously track the movements of multiple animals within the geographic area of a network. Our approach of using filters to limit which nodes are used in RSS‐based localization estimates greatly improved accuracy, but further improvement in location accuracy could be gained by coupling other analytical strategies with our methods. For example, leveraging the autocorrelation of a sequence of locations through time (e.g., lack of independence of previous and subsequent locations) could reduce outlier location estimates by constraining estimates to biologically plausible outcomes based on information from successive locations (Baktoft et al., 2017; Fleming et al., 2016). Additionally, non‐linear localization techniques could be used to accommodate noise in an environment by mapping, or fingerprinting, signal strength patterns across an entire network and then using the resulting map of radio signals for each location to train machine learning algorithms to estimate locations (Harbicht et al., 2017). Location fingerprinting has been widely used in indoor wireless networks and can increase localization accuracy by accounting for the effects of obstructions or bounce in a radio signal map, along with variation in receiver sensitivity (Campos & Lovisolo, 2019). However, the complexity of such an approach may only be worthwhile in long‐term networks that can be carefully monitored to ensure consistency of signal patterns across the node network. Moreover, other localization algorithms, such as time‐difference‐of‐arrival (TDOA), can also be applied to data collected from node networks and may increase the accuracy of localization estimates over RSS‐based methods (Baktoft et al., 2017; MacCurdy et al., 2019; Piersma et al., 2014). However, TDOA‐based localization methods, which estimate a signal’s position based on differences in the time of arrival of a signal at each receiver, require receivers to be synchronized and capable of measuring time at least to the millisecond, increasing the complexity, cost, and energy requirements of receivers (Buehrer & Venkatesh, 2019). Ultimately, the level of accuracy in location estimates scales the research questions that can be asked. As the technology and analytical approaches continue to be developed, and as experience with node networks increase, the use of these type of automated radio tracking systems will likely increase in ecological research and allow for many important ecological questions to be explored.

## CONFLICT OF INTEREST

The authors have no conflict of interest to declare.

## AUTHOR CONTRIBUTION

**Kristina L. Paxton:** Conceptualization (equal); Data curation (lead); Formal analysis (lead); Methodology (equal); Writing – original draft (lead); Writing – review & editing (equal). **Kayla M. Baker:** Investigation (equal); Writing – review & editing (supporting). **Zia B. Crytser:** Investigation (equal); Writing – review & editing (supporting). **Ray Mark P. Guinto:** Investigation (equal); Writing – review & editing (supporting). **Kevin W. Brinck:** Formal analysis (supporting); Writing – review & editing (supporting). **Haldre S. Rogers:** Conceptualization (equal); Project administration (equal); Writing – review & editing (supporting). **Eben H. Paxton:** Conceptualization (equal); Methodology (equal); Project administration (equal); Writing – original draft (supporting); Writing – review & editing (supporting).

## Data Availability

Data used for field‐based tests are available via a U.S. Geological Survey ScienceBase data release, https://doi.org/10.5066/P94LQWIE (Paxton et al., 2022). R code used for simulations, downloading raw RSS values from the CTT servers for test data, and processing field‐based test datasets are available via KLP’s GitHub account: https://github.com/kpaxton75.
